# Efficacy, Toxicity and Effect of Pretreatment Cardiologic Consultation on Outcomes of Ibrutinib Therapy for Chronic Lymphocytic Leukemia—A KroHem Study

**DOI:** 10.3390/cancers17142302

**Published:** 2025-07-10

**Authors:** Inga Mandac Smoljanović, Igor Aurer, Nikola Bulj, Barbara Dreta, Antonija Miljak, Fran Petričević, Marija Ivić, Sandra Bašić-Kinda, Viktor Zatezalo, Sanja Madunić, Dubravka Čaržavec, Jasminka Sinčić-Petričević, Dragana Grohovac, Ozren Jakšić, Ivan Krečak, Martina Morić-Perić, Božena Coha, Petra Berneš, Neno Živković, Vlatko Pejša

**Affiliations:** 1 University Hospital Merkur, 10000 Zagreb, Croatia; imandac@yahoo.com (I.M.S.); viktor.zatezalo@gmail.com (V.Z.); 2 University Hospital Centre Zagreb, 10000 Zagreb, Croatia; bdreta@gmail.com (B.D.); sandra.kinda@gmail.com (S.B.-K.); nenozivkovic12@gmail.com (N.Ž.); 3 School of Medicine, University of Zagreb, 10000 Zagreb, Croatia; nikolabulj@gmail.com (N.B.); ojaksic@kbd.hr (O.J.); 4 University Hospital Centre Sisters of Mercy, 10000 Zagreb, Croatia; fran.5tricevic@gmail.com (F.P.); dcarzavec@gmail.com (D.Č.); 5 University Hospital Centre Split, 21000 Split, Croatia; antonija.miljak003@gmail.com (A.M.); sanja.madunic@windowslive.com (S.M.); 6 University Hospital Dubrava, 10000 Zagreb, Croatia; marijai559@gmail.com (M.I.);; 7 University Hospital Centre Osijek, 31000 Osijek, Croatia; sincicpetricevic@yahoo.com; 8 University Hospital Centre Rijeka, 51000 Rijeka, Croatia; dzdrijeka@gmail.com; 9 School of Medicine, University of Rijeka, 51000 Rijeka, Croatia; krecak.ivan@gmail.com; 10 General Hospital Šibenik, 22000 Šibenik, Croatia; 11 General Hospital Zadar, 23000 Zadar, Croatia; martina.moricperic@gmail.com; 12 General Hospital Dr. Josip Benčević, 35000 Slavonski Brod, Croatia; bcoha59@gmail.com; 13 General Hospital Pula, 52100 Pula, Croatia; petrabernes@yahoo.com

**Keywords:** chronic lymphocytic leukemia, ibrutinib, efficacy, toxicity, cardiologic evaluation

## Abstract

**Background/Objectives:** Ibrutinib has revolutionized the treatment of chronic lymphocytic leukemia but has off-target side effects, most notably cardiac. In order to evaluate the efficacy and toxicity of ibrutinib treatment, risk factors for adverse outcomes and the influence of pretreatment cardiologic evaluation, KroHem collected data on Croatian patients with chronic lymphocytic leukemia treated with this drug. **Methods:** This is a retrospective survey performed in order to analyze the efficacy and toxicity of ibrutinib in a real-life setting. Patients starting therapy with ibrutinib for chronic lymphocytic leukemia between the time the drug became reimbursable in 2015 and 31 December 2021 were included, irrespective of treatment line. **Results**: We identified 436 patients fulfilling entry criteria; 404 (92.7%) responded to treatment. Cardiovascular side effects occurred in 25.0% of patients and hemorrhagic in 15.6%. The dose of ibrutinib was permanently reduced in 22.2% of patients. Median follow-up of the cohort was 29 months (IQR 18–41 months), estimated median overall survival 75 months (IQR 36 months–not reached), progression-free survival 54 months (IQR 24–81 months) and time on ibrutinib treatment 44 months (IQR 14–78 months). Factors significantly related to overall survival in multivariate analysis were stage, treatment line and age. Factors significantly related to progression-free survival in multivariate analysis were treatment line, age and pretreatment history or ECG finding of cardiac arrhythmia. Factors significantly related to time on ibrutinib treatment in multivariate analysis were age, pretreatment history or ECG finding of cardiac arrhythmia, and permanent dose reduction for toxicity. Sex, FISH and the presence of arterial hypertension were not independently significantly related to any of these outcomes. Pretreatment cardiologic consultation did not improve time on ibrutinib therapy, progression-free survival, overall survival, risk of stopping treatment due to cardiovascular side effects or risk of cardiovascular or sudden death, neither in the whole cohort nor in the subgroup of patients with and without pretreatment cardiac arrhythmia. **Conclusions**: Our analysis confirms the efficacy and tolerability of ibrutinib for the treatment of chronic lymphocytic leukemia. Patients older than 75 do significantly less well. Routine pretreatment cardiologic consultation does not improve outcomes and should not be considered part of standard pretreatment assessment without additional proof of its usefulness. Future investigations should aim at identifying predictive factors, mechanisms, and preventive strategies for reducing cardiotoxicity in chronic lymphocytic leukemia patients taking Bruton tyrosine kinase inhibitors.

## Simple Summary

We analyzed the efficacy and toxicity of ibrutinib and risk factors for adverse outcomes in 436 patients with chronic lymphocytic leukemia. The response rate was 92.7%. Cardiovascular side effects occurred in 25.0% of patients and hemorrhagic in 15.6%. The dose of ibrutinib was permanently reduced in 22.2% of patients. Median overall survival was 75 months (interquartile range (IQR) 36 months–not reached), progression-free survival 54 months (IQR 24–81 months) and time on treatment 44 months (IQR 14–78 months). Factors significantly related to overall survival were stage, treatment line and age. Factors significantly related to progression-free survival were treatment line, age and history or ECG finding of arrhythmia. Pretreatment cardiologic consultation did not improve efficacy nor reduce toxicity. Our analysis confirms the efficacy and tolerability of ibrutinib. Patients older than 75 do significantly less well. Routine pretreatment cardiologic consultation does not improve outcomes.

## 1. Introduction

Ibrutinib is the first small-molecule Bruton tyrosine kinase inhibitor approved for treatment of chronic lymphocytic leukemia, initially for relapsed/refractory disease and patients with del 17p, and later also for frontline treatment irrespective of genetic features [1,2,3]. A series of clinical trials showed ibrutinib to be superior to immunochemotherapy in all studied settings. This resulted in ibrutinib becoming the most prescribed drug for CLL treatment.

Ibrutinib is generally well tolerated but, despite the fact that it is a targeted agent, has off-target side effects. Probably the most important are cardiovascular, including hypertension and arrhythmias, most frequently atrial fibrillation [4]. Other side effects include bleeding, muscular and articular pain and skin reactions [3,4,5,6,7,8].

A substantial number of patients enrolled in clinical trials had to stop treatment for toxicity, mainly cardiac [8,9]. Therefore, some experts suggest performing cardiologic evaluation prior to treatment start [9,10]. However, the usefulness of this approach has not been studied.

Ibrutinib became reimbursable in Croatia in 2015, first for patients refractory to or in early relapse after immunochemotherapy and those with del 17p, and later for all front-line patients in need of therapy. In some centers, pretreatment cardiac consultation was considered a standard of care and performed routinely. In order to evaluate the efficacy and toxicity of ibrutinib treatment, risk factors for adverse outcomes and the effect of pretreatment cardiologic consultation in a real-life setting, KroHem, the Croatian Cooperative Group for Hematological Diseases, collected and analyzed data on all Croatian patients receiving ibrutinib for chronic lymphocytic leukemia.

## 2. Materials and Methods

Patients were included in the study if they had chronic lymphocytic leukemia diagnosed according to standard criteria and started ibrutinib treatment between 2015 and 2021. Data on patient demographics (age and sex), history (previous treatment, history of arterial hypertension or cardiac arrhythmias), baseline characteristics (presence of arterial hypertension, ECG finding of cardiac arrhythmia, pretreatment cardiologic evaluation, Binet stage, interphase fluorescence in situ hybridization (FISH) and/or presence of p53 mutations, immunoglobulin heavy chain mutational status) and outcomes (overall survival, progression-free survival, time on ibrutinib treatment, side effects, permanent ibrutinib dose reduction, reasons for stopping treatment, cause of death) were retrieved from hospital records. All patients had a pretreatment ECG. Of those who underwent cardiologic consultation, 95% had cardiac ultrasound and 55% had a 24 h Holter ECG.

Response to treatment was evaluated according to the relevant International Workshop Group on Chronic Lymphocytic Leukemia response criteria [11]. Overall survival was defined as the period from the date of ibrutinib initiation to death irrespective of cause. Progression-free survival was defined as the period from the date of ibrutinib initiation to treatment failure (including insufficient response and progression) or death. Time on ibrutinib treatment was defined as the period from ibrutinib initiation until permanent treatment stop irrespective of cause. Descriptive statistics, available within the MicroSoft Excel 2016^®^ program, were used to summarize baseline characteristics. Survival curves were plotted using the Kaplan–Meier method, and their differences were compared with the log-rank test using SPSS v. 26 (IBM, Amonk, NY, USA). Variables, found to be statistically significant prognostic factors at level *p* < 0.05 in univariate analysis, were included in a multivariate analysis to identify those that would retain their statistical significance in a forward regression model using the same program.

The study was performed in accordance with all pertaining Croatian, European Union and international rules and regulations and with the approval of the Ethical Committee of the University Hospital Merkur, Zagreb, Croatia, number 03/1-2956. Since this was a retrospective study of anonymized patient data, a waiver of the requirement for informed consent was obtained.

## 3. Results

We identified 436 patients fulfilling entry criteria (Table 1). The median age was 68, with a range of 36–87 years. The majority of patients were male (268, 61.4%); 10.6% had Binet stage A, 48.3% B and 41.1% C. FISH results were available for 81% of patients; 32.1% had del 17p and/or p53 mutations. Immunoglobulin heavy chain mutational status was known in only 14% of patients. Almost half (49.5%) received ibrutinib as a frontline treatment, approximately a quarter (26.6%) in the second, and a similar number (23.9%) in the third or later therapy line. All patients receiving ibrutinib in the second or later lines of therapy were previously treated with immunochemotherapy, and none have received targeted agents prior to ibrutinib. Pretreatment cardiologic consultation before initiating treatment was performed in 30.3% of patients; 12.4% had a history or ECG finding of cardiac arrhythmia, and 50.2% had arterial hypertension. Of the 54 patients with arrhythmias, one had a pacemaker inserted for third-degree AV block, two had a history of supraventricular paroxysmal tachycardia, and 51 had atrial fibrillation.

At the time of data cutoff, 312 patients (71.6%) were alive, 263 (60.3%) were progression-free, and 233 (53.4%) were still on ibrutinib therapy.

### 3.1. Toxicity

A total of 34 patients (7.8%) died after progression, 18 (4.1%) suddenly or of cardiovascular causes, 4 (0.9%) of bleeding, 13 (3.0%) of other tumors, 16 (3.7%) of COVID-19, 20 (4.6%) of other infections, and 2 frail elderly (0.5%) after general deterioration, and in 17 (3.9%) the cause of death is unknown.

Ibrutinib was stopped in 60 (13.8%) patients for insufficient response or progression, of whom 19 had Richter’s syndrome; in 7 (1.6%), for patient decision; in 6 (1.4%), for treatment of another disease; in 75 (17.2%), due to intercurrent death; and in 55 (12.6%), for toxicity, specifically cardiovascular 26, bleeding 8, gastrointestinal 5, cutaneous 5, neurologic 3, hematologic 3, infections 3, and pneumonitis and ocular 1 each.

Cardiovascular side effects occurred in 25.0% of patients and hemorrhagic in 15.6%. In the group without previous cardiac arrhythmias, 10% developed atrial fibrillation and 13% other cardiac side effects, mostly arterial hypertension. In the group with previous cardiac arrhythmias, 22% developed atrial fibrillation and 16% other cardiac side effects. The dose of ibrutinib was permanently reduced in 22.2% of patients.

### 3.2. Efficacy

**Figure 1:**
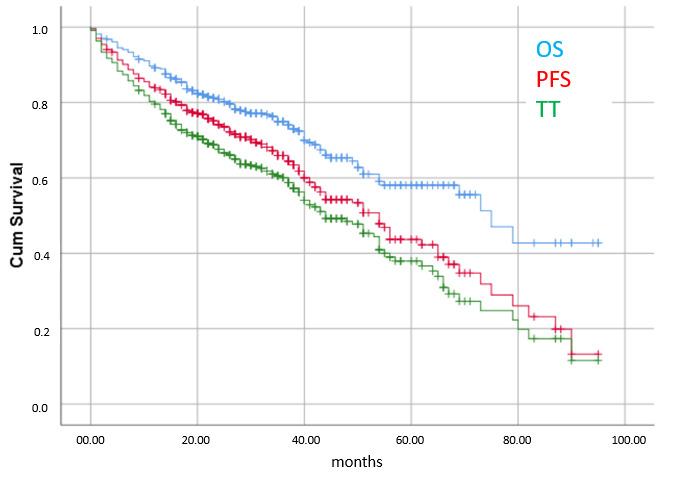
Overall survival (OS), progression-free survival (PFS) and time on ibrutinib therapy (TT) of the whole cohort.

The response rate was 92.7%. Median follow-up was 29 months, with a range of 1–95 months and an IQR of 18–41 months. Estimated median overall survival was 75 months (IQR 36 months—not reached), and estimated progression-free survival was 54 months (IQR 24–81 months). Estimated median time on ibrutinib therapy was 44 months (IQR 14–78 months) (Figure 1).

### 3.3. Prognostic Factors

**Figure 2:**
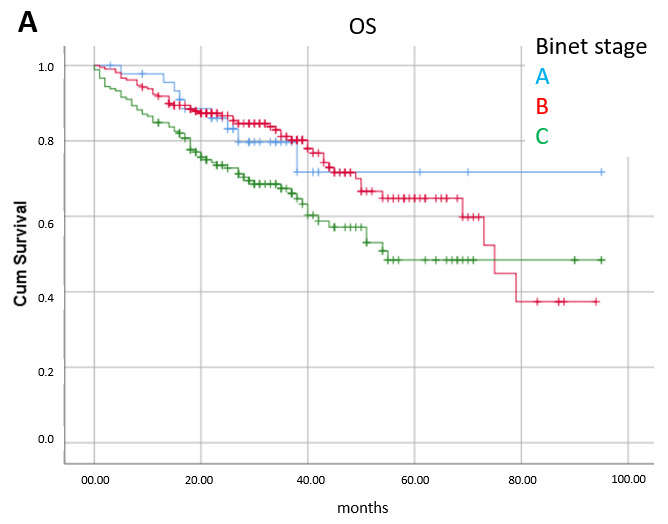

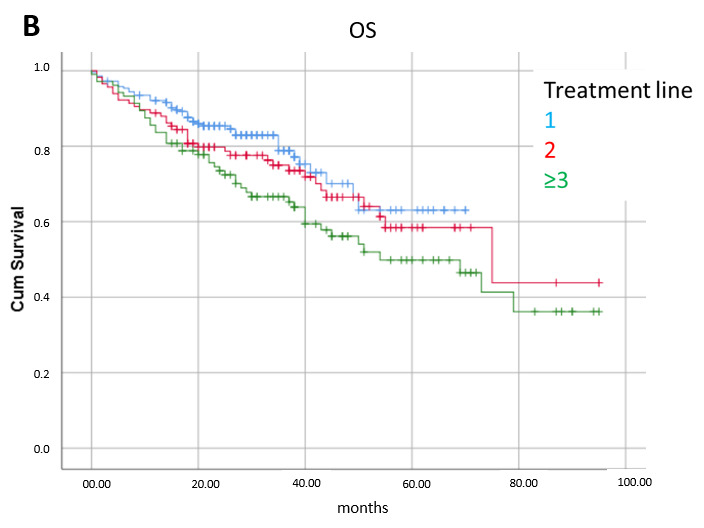

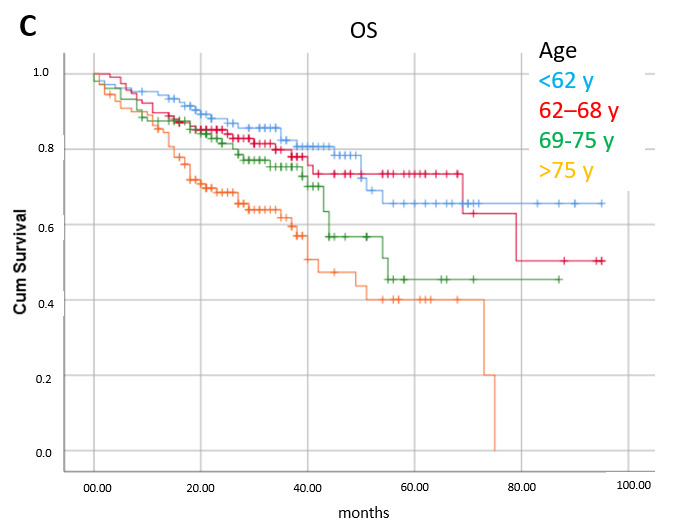
Overall survival (OS) according to prognostic factors significant in multivariate analysis: (**A**) disease stage (*p* = 0.009), (**B**) treatment line (*p* = 0.036) and (**C**) age (*p* < 0.0001).

Factors significantly related to overall survival in multivariate analysis were stage (Binet A: median OS not reached, B: 75 mo, C: 55 mo, *p* = 0.009); treatment line (first line: median not reached, second line: median 75 mo, ≥3rd line: median 54 mo, *p* = 0.036); and age (≤68 y: median not reached, 68–75 y: 55 mo, >75 y: 42 mo, *p* < 0.0001). (Figure 2).

**Figure 3:**
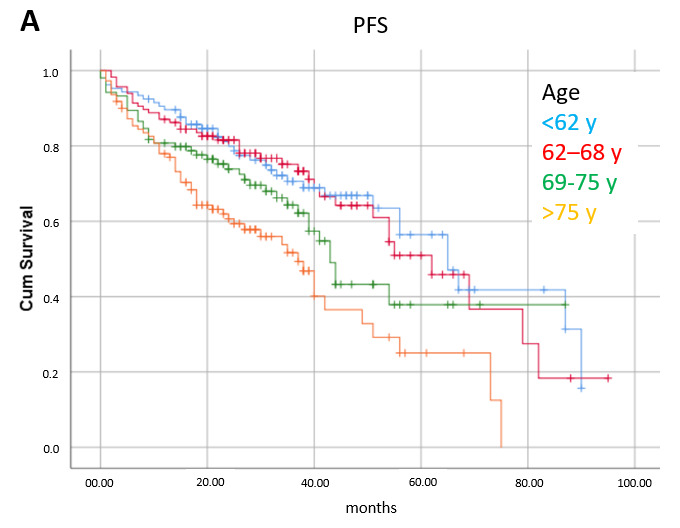

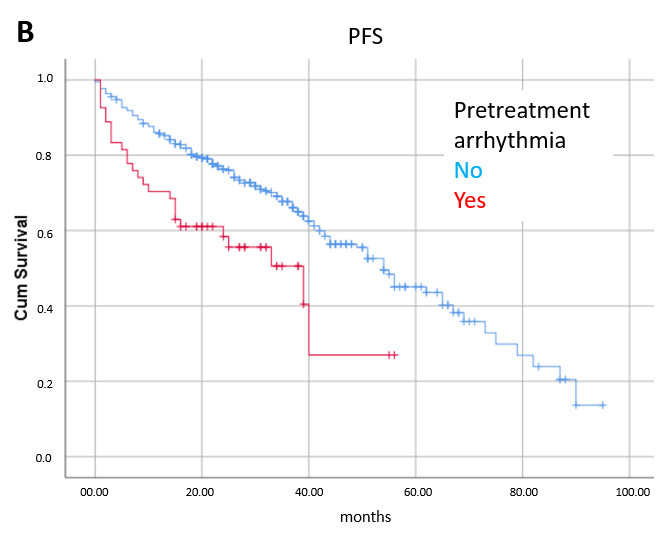

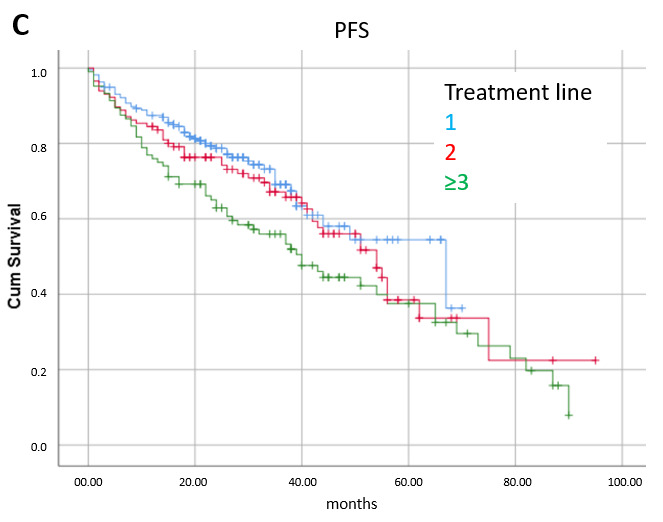
Progression-free survival (PFS) according to prognostic factors significant in multivariate analysis: (**A**) age (*p* < 0.0001), (**B**) pretreatment history or ECG finding of cardiac arrhythmia (*p* = 0.001), (**C**) treatment line (*p* = 0.023).

Factors significantly related to progression-free survival in multivariate analysis were age (≤68 y: median 64 mo, 68–75 y: 43 mo, >75 y: 37 mo, *p* < 0.0001); pretreatment history or ECG finding of cardiac arrhythmia (no: 54 mo, yes: 39 mo, *p* = 0.001); and treatment line (first line: median 67 mo, second: 54 mo, ≥ third: 40 mo, *p* = 0.023) (Figure 3).

**Figure 4:**
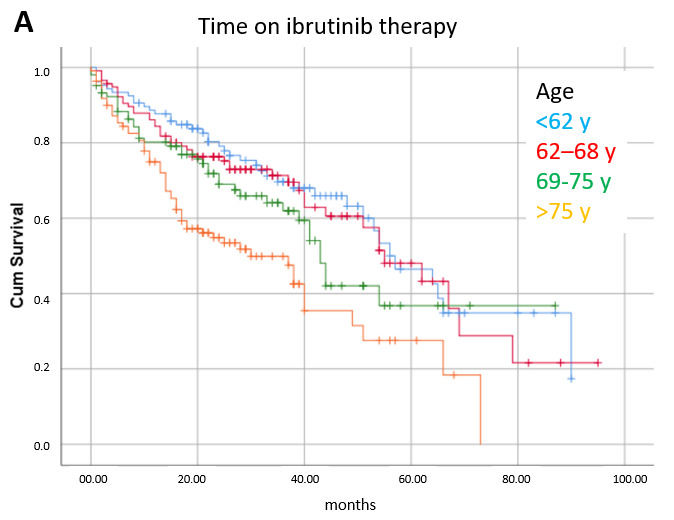

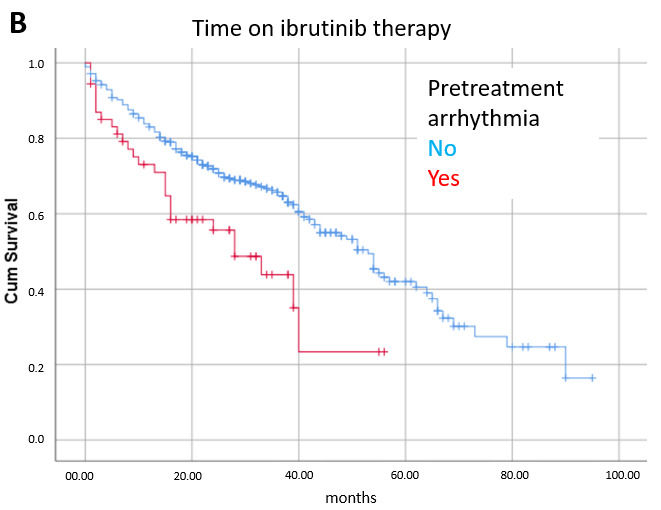

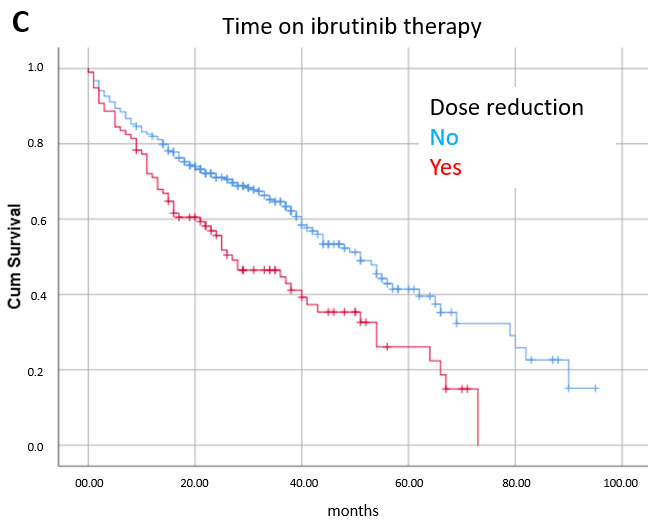
Time on ibrutinib therapy according to prognostic factors significant in multivariate analysis: (**A**) age (*p* < 0.0001), (**B**) pretreatment history or ECG finding of cardiac arrhythmia (*p* < 0.0001), (**C**) permanent dose reduction for toxicity (*p* < 0.0001).

Factors significantly related to time on ibrutinib therapy in multivariate analysis were age (≤68 y: median 56 mo, 68–75 y: 41 mo, >75 y: 25 mo, *p* < 0.0001), pretreatment cardiac arrhythmia (no: 51 mo, yes: 24 mo, *p* < 0.0001), and need for permanent ibrutinib dose reduction (no: median 51 mo, yes: 27 mo, *p* < 0.0001) (Figure 4).

Sex, FISH and the presence of arterial hypertension were not independently significantly related to any of these outcomes.

**Figure 5:**
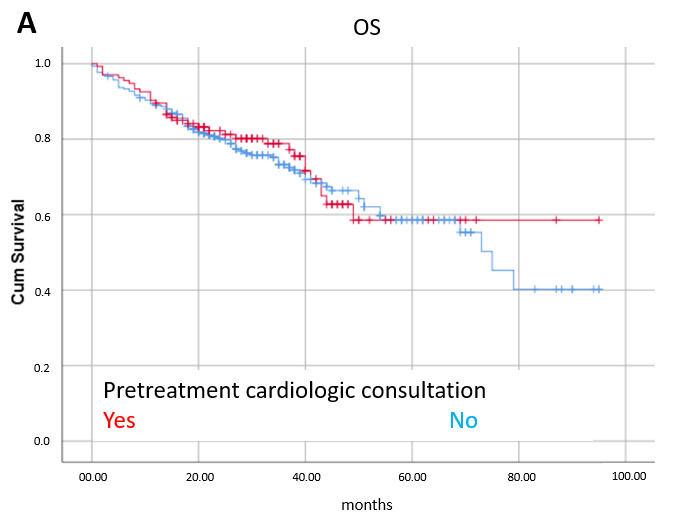

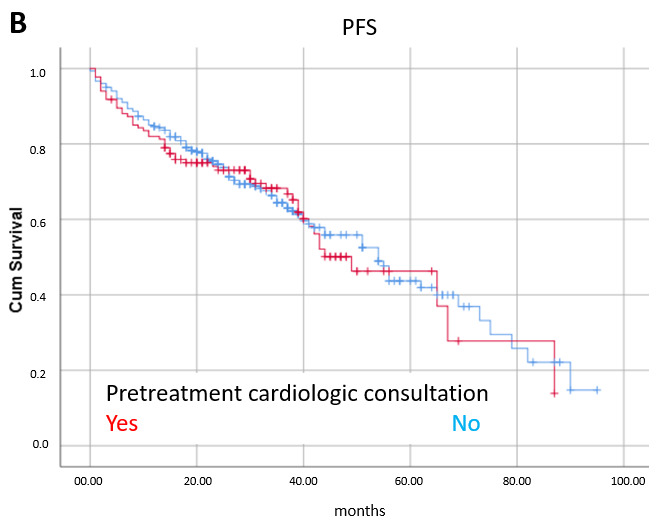

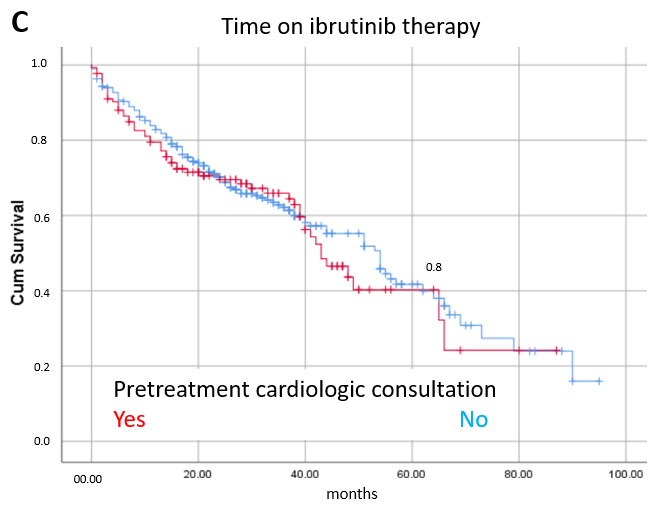
(**A**) Overall survival (OS), (**B**) progression-free survival (PFS) and (**C**) time on ibrutinib therapy according to pretreatment cardiologic consultation.

Pretreatment cardiologic consultation did not improve overall survival, progression-free survival, time on ibrutinib therapy, the risk of stopping treatment due to cardiovascular side effects or the risk of cardiovascular or sudden death, neither in the whole cohort nor in the subgroups of patients with or without pretreatment cardiac arrhythmia. (Figure 5).

## 4. Discussion

This is a real-life, non-interventional, retrospective analysis of patients starting treatment with ibrutinib for chronic lymphocytic leukemia between 2015 and 2021. The cohort is very probably comprehensive, because all Croatian hematology centers using ibrutinib participated, and, due to reimbursement rules, it was easy to identify all treated patients even retrospectively. However, as with any such cohort, treatment decisions might have been influenced by unrecognized factors, and conclusions on causality are therefore difficult to make.

While data on FISH are available for most of the patients, immunoglobulin heavy chain mutational status is known for only a minority, since this technique became available in Croatia only in 2021. We therefore did not analyze the influence of immunoglobulin heavy chain mutational status on outcomes. However, results of clinical studies suggest that this factor does not significantly impact outcomes of treatment with Bruton tyrosine kinase inhibitors [12].

In our cohort, del 17p was present in a higher percentage of patients than in other series, especially in previously untreated patients. This was due to reimbursement rules; during most of the enrollment period, ibrutinib was not reimbursable for frontline treatment of patients who did not have del 17p or p53 mutation. This enrollment bias probably explains the lack of prognostic importance of FISH in our and other real-world series [13,14,15,16,17].

The outcome of our patients receiving ibrutinib for relapsed or refractory chronic lymphocytic leukemia was similar to that in other published real-life series [5,13,14,15,16,17,18,19,20,21,22,23], while the outcome of frontline therapy seems somewhat inferior to that reported in large clinical trials [7,8,24]. Again, this is probably the consequence of an overrepresentation of high-risk patients in the frontline series due to reimbursement rules, as explained earlier, and the inclusion of unfit patients with significant comorbidities. Richter’s syndrome occurred in 4.4% of all and 31.7% of progressing patients, confirming it as a major obstacle to successful chronic lymphocytic leukemia therapy in patients receiving targeted agents.

Age was the most important prognostic factor for all analyzed outcomes. It had a threshold effect, with patients between 62 and 68 having similar outcomes as those younger than 62 (two youngest quartiles). In contrast, those older than 75 (oldest quartile) had the worst prognosis. Also, patients with Binet stage A and B had similar outcomes, while those with stage C fared worse. Patients receiving ibrutinib in their first or second line of treatment had similar outcomes, while those treated in later lines fared worse. The latter finding might be clinically interesting for choosing between time-limited and continuous frontline therapy options. Most of the published real-life series did not analyze prognostic factors in great detail, but some found age [19,21], stage [16,18] and number of previous treatment lines [14,16,18] to be of prognostic significance.

Regarding tolerability, as in most other published series [5,13,14,18,19,21], we found that toxicity, foremost cardiovascular, is the major cause of ibrutinib treatment interruption. Patients progressed a median of 10 months after stopping ibrutinib. This suggests that, in patients who achieved a response to Bruton tyrosine kinase inhibitors, their effect persists for some months after they are stopped and that short interruptions in this situation are not necessarily harmful. The sudden and cardiac death rate in our series was 4%, which is similar to results of major clinical trials. The rates reported in real-life series are very different and range from 0 [22] to 5% [23], probably due to differences in patient selection and length of follow-up. The major cause of death in non-progressing patients was infections. Approximately 8% died of infection, slightly less than half of COVID-19, which is not surprising, since the pandemic was ravaging during much of the follow-up. More than half of infection-related deaths were caused by other agents, serving as a reminder of the immunosuppressive effect of chronic lymphocytic leukemia (and, to a lesser degree, Bruton tyrosine kinase inhibition).

More than half of our patients had arterial hypertension at the start of treatment. These patients did not fare worse than those without hypertension, at least not in multivariate analysis. Similar results were seen in the cohort from the MD Anderson Cancer Center [25]. Our series also confirms findings from other studies that reducing the dose of ibrutinib for toxicity does not negatively influence progression-free survival and overall survival [14,26].

The fact that pretreatment cardiologic evaluation failed to reduce cardiac toxicity is a sobering reminder of the complexity of the cardiac effects of ibrutinib and the liability of imposing simple untested solutions to complicated clinical problems. Ibrutinib has been related to multiple cardiovascular complications, including atrial fibrillation, arterial hypertension, ventricular tachycardia, atrioventricular dissociation, and heart failure [27]. Previous studies have indicated that treatment with ibrutinib is associated with a 5 to 20% increase in the incidence of atrial fibrillation [13,28]. Atrial fibrillation is fairly common among the general population, but patients with cancer have elevated prevalence and incidence of atrial fibrillation compared with the general population. A nationwide 12-year cohort study in Denmark reported that patients with cancer had an atrial fibrillation incidence of 17.4 per 1000 person-years compared with 3.7 per 1000 person-years in patients without cancer, with the risk of new-onset atrial fibrillation highest in the first 90 days after cancer diagnosis [29].

In a group of 860 chronic lymphocytic leukemia patients treated with ibrutinib at the Padua University Hospital, 47 (5.6%) developed atrial fibrillation after a median follow-up of 9.4 years. Variables associated with an increased risk of atrial fibrillation were age > 65 y, male sex, valvular heart disease, cardiomyopathy, hypo/hyperthyroidism, chronic lung disease, type 2 diabetes mellitus and grade 3 infections [30]. One explanation why cardiologic evaluation was not useful is that most of these risk factors are easily identified by hematologists, who have basic training in other internal medicine fields, including cardiology. Cardiologists can additionally identify the very rare patient with asymptomatic cardiomyopathy, but they are too few to significantly improve the outcome of the whole cohort. It seems that most of the patients who developed cardiac side effects during ibrutinib therapy did not have any known and identifiable risk factors or, at the time they started treatment, did not have a valid therapeutic alternative (e.g., those with a history or pretreatment finding of arrhythmia in our series). This might be different nowadays, with the introduction of other Bruton tyrosine kinase inhibitors, especially acalabrutinib, which has fewer cardiac side effects [31]. Cardiologic consultation should therefore probably be sought only in patients in whom treatment with Bruton tyrosine kinase inhibitors is considered the best possible option but who have signs and symptoms of possible cardiac disease.

## 5. Conclusions

In conclusion, our analysis confirms the efficacy and tolerability of ibrutinib for the treatment of chronic lymphocytic leukemia. Patients older than 75 do significantly less well. Those with pretreatment cardiac arrhythmias are at an increased risk of having to stop treatment early and should probably be offered different therapies. Routine pretreatment cardiologic consultation does not seem to improve outcomes and should not be considered part of standard pretreatment assessment without additional proof of its usefulness. Future investigations should aim at identifying predictive factors, mechanisms, and preventive strategies for reducing cardiotoxicity in chronic lymphocytic leukemia patients taking Bruton tyrosine kinase inhibitors.

## Figures and Tables

**Table 1:** Patient characteristics.

Patient’s Characteristic	No. (%)
Sex: M/F	268 (68.5%)/168 (31.5%)
Age (median/range/IQR)	68 years/36–87 years/62–76 years
Binet stage * (A/B/C)	46 (10.6%)/209 (48.3%)/178 (41.1%)
FISH: not performed	85 (19%)
FISH: normal/del 11/+12/del 13/del 17	112 (31.0%)/49 (13.6%)/22 (6.1%)/62 (17.2%)/116 (32.1%)
Immunoglobulin heavy chain mutation: not analyzed	378 (86%)
Immunoglobulin heavy chain: mutated/unmutated	10 (17%)/48 (83%)
Prior lines of treatment: 0/1/≥2	216 (49.5%)/116 (26.6%)/104 (23.9%)
	
Prior lines of treatment: (range)	0–10
Cardiology consultation before ibrutinib ** (yes/no)	132 (30.3%)/303 (69.7%)
History or ECG finding of cardiac arrhythmia before ibrutinib ** (yes/no)	54 (12.4%)/381 (87.6%)
	
Arterial hypertension before ibrutinib (yes/no)	219 (50.2%)/217 (49.8%)

IQR = interquartile range; * unknown for 3 patients; ** unknown for 1 patient.

## Data Availability

Due to the informed consent waiver, any requests for original data should be sent to the corresponding author and must be approved by a Croatian Ethics Committee.
